# Antibody Recognition of Cancer-Related Gangliosides and Their Mimics Investigated Using *in silico* Site Mapping

**DOI:** 10.1371/journal.pone.0035457

**Published:** 2012-04-20

**Authors:** Mark Agostino, Elizabeth Yuriev, Paul A. Ramsland

**Affiliations:** 1 Medicinal Chemistry and Drug Action, Monash Institute of Pharmaceutical Sciences, Monash University, Parkville, Victoria, Australia; 2 Centre for Immunology, Burnet Institute, Melbourne, Victoria, Australia; 3 Department of Surgery Austin Health, University of Melbourne, Heidelberg, Victoria, Australia; 4 Department of Immunology, Monash University, Alfred Medical Research and Education Precinct, Melbourne, Victoria, Australia; University of Queensland, Australia

## Abstract

Modified gangliosides may be overexpressed in certain types of cancer, thus, they are considered a valuable target in cancer immunotherapy. Structural knowledge of their interaction with antibodies is currently limited, due to the large size and high flexibility of these ligands. In this study, we apply our previously developed site mapping technique to investigate the recognition of cancer-related gangliosides by anti-ganglioside antibodies. The results reveal a potential ganglioside-binding motif in the four antibodies studied, suggesting the possibility of structural convergence in the anti-ganglioside immune response. The structural basis of the recognition of ganglioside-mimetic peptides is also investigated using site mapping and compared to ganglioside recognition. The peptides are shown to act as structural mimics of gangliosides by interacting with many of the same binding site residues as the cognate carbohydrate epitopes. These studies provide important clues as to the structural basis of immunological mimicry of carbohydrates.

## Introduction

Gangliosides are glycosphingolipids which feature one or more sialic acid residues. They are most often associated with nervous system function, where they play a crucial role in maintaining the stability of myelin and axons [1]. Alterations in ganglioside expression levels have been associated with several neurodegenerative conditions, including Alzheimer's disease, Parkinson's disease, Huntington's disease and HIV-associated dementia [2]. The production of anti-ganglioside antibodies is one of the key biochemical features of Guillain-Barré syndrome, an autoimmune neuropathy [3]. While the specific cause of the syndrome is unknown in the majority of cases, it is commonly preceded by infection with *Campylobacter jejuni* [4], [5].

**Figure 1:**
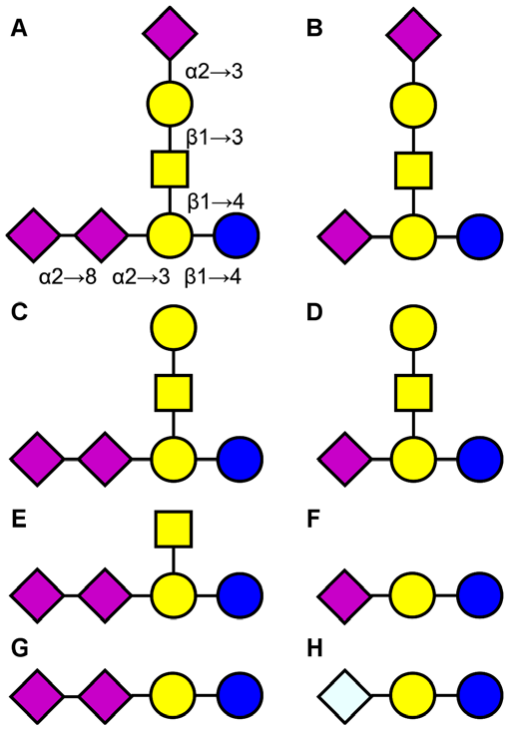
Carbohydrate determinants of gangliosides commonly found in the central nervous system and cancer cells. CNS gangliosides: A. GT1b. B. GD1a. C. GD1b. D. GM1. Cancer-related gangliosides: E. GD2. F. GD3. G. GM3. H. Neu5Gc-GM3. Since each structure is differentiated by the removal of one or more residues from GT1b, the glycosidic linkages specified on GT1b apply to all of the structures, including Neu5Gc-GM3. Carbohydrate symbols follow the nomenclature of the Consortium for Functional Glycomics [63]: *N*-acetylneuraminic acid – purple diamond; galactose – yellow circle; *N*-acetylgalactosamine – yellow square; glucose – blue circle; *N*-glycolylneuraminic acid – light blue diamond.

Gangliosides have been identified as tumor-associated carbohydrate antigens (TACAs), a group which includes Lewis Y, Lewis X, Thomsen-Friedenreich and Thomsen-nouvelle [6]. The gangliosides most often found in the nervous system are GM1, GD1a, GD1b and GT1b [1], however, the gangliosides considered for vaccine and antibody-based targeting are generally biosynthetic intermediates of these, such as GM2, GM3, GD2 and GD3 [7], [8], [9], [10] (Figure 1). While found in low amounts in the nervous system, they often appear in high densities on a variety of tumor cell types [11], [12], [13]; thus, they are attractive targets for cancer immunotherapy. In addition to these, gangliosides terminating in *N*-glycolylneuraminic acid (Neu5Gc), such as *N*-glycolyl GM3 (Neu5Gc-GM3), are also useful targets for cancer treatment [14], [15]. Unlike *N*-acetylneuraminic acid, Neu5Gc cannot be synthesized by humans, due to the lack of a functional sialic acid hydroxylase [16]. The appearance of Neu5Gc on human cells is thought to come about through enzymatic incorporation from diet [17]. Since Neu5Gc expression is largely restricted to cancer cells in humans, targeting Neu5Gc-terminating gangliosides is likely to achieve a highly selective therapeutic outcome.

Carbohydrates are normally considered T cell-independent antigens, typically incapable of inducing a strong immune response [18], [19]. One way to address this issue is through the development of carbohydrate mimetics, capable of inducing an anti-carbohydrate immune response. Peptides have been considered for this purpose against a wide range of targets [20]. Peptide mimics of the GD2 [21], [22], [23] and GD3 [24], [25] gangliosides have been identified, typically by phage display against anti-ganglioside antibodies. Some of these have been found to induce anti-ganglioside immune responses [23], [25], [26]. Peptide mimics of anti-Neu5Gc-GM3 antibodies are not currently known, however, anti-idiotypic antibodies against these antibodies have been identified [27], [28].

Although prior structural studies into the recognition of gangliosides and their mimics by antibodies have been performed [21], [29], [30], [31], these have generally utilized simple molecular docking methodologies. We have previously developed the site mapping technique, which we have demonstrated to be effective for studying carbohydrate-antibody [32], [33] and carbohydrate-lectin recognition [34], as well as peptide-antibody recognition [35], [36], [37], [38]. Here, we evaluate a range of molecular docking programs for their ability to predict the binding modes of acidic sugars to antibodies and apply our site mapping technique to study antibody recognition of acidic sugars (Table 1). The computational approach most suitable for the validation cases is then utilized to investigate recognition of carbohydrate epitopes of gangliosides by four anti-ganglioside antibodies: R24, ME36.1, chP3 and 14F7 (Table 2). While there are experimentally solved native structures of all of the antibodies of interest available, the structure of one of these antibodies, chP3, is missing a short segment of the key HCDR3 loop. We have thus extended the site mapping technique to consider multiple protein conformers, in a process termed “dynamic site mapping”. Finally, the site mapping technique is used to investigate antibody recognition of ganglioside-mimetic peptides, which is compared to recognition of the carbohydrate determinants of gangliosides.

**Table 1:** Validation systems.

PDB ID	Carbohydrate	Antibody	Resolution	Reference
1Q9Q	Kdoα(2→8)Kdoα(2→4)Kdoα(2-OAll)	S25-2	1.49	[50]
1Q9T	Kdoα(2→8)Kdoα(2-OAll)	S25-2	1.74	[50]
3HZK	Kdoα(2→4)Kdoα(2-OAll)	S73-2	2.15	[51]
3HZV	Kdoα(2→8)Kdoα(2→4)Kdoα(2-OAll)	S73-2	1.90	[51]
3HZY	Kdoα(2→4)Kdoα(2→4)Kdoα(2-OAll)	S73-2	2.10	[51]
3OKK	Kdoα(2→4)Kdoα(2-OAll)	S25-39	1.95	[52]
3OKL	Kdoα(2→8)Kdoα(2-OAll)	S25-39	1.80	[52]
3OKN	Kdoα(2→4)Kdoα(2→4)Kdoα(2-OAll)	S25-39	2.15	[52]
3OKO	Kdoα(2→8)Kdoα(2→4)Kdoα(2-OAll)	S25-39	2.45	[52]

**Table 2:** Test systems.

PDB ID	Antibody	Carbohydrate	Mimic^a^	Resolution (Å)	Reference
1BZ7	R24	GD3	RHAYRSMAEWGFLYS	2.50	[66]
1PSK	ME36.1	GD2	LDVVLAWRDGLSGAS	2.80	[67]
1RIH	14F7	Neu5Gc-GM3	mAb 4G9	2.50	[29]
3IU4	chP3	Neu5Gc-GM3	mAb 1E10	1.75	[30]

a Only peptide-based inhibitors are examined in the current study. Anti-idiotypic antibodies are included for reference.

## Methods

### Validation and test systems

For method validation, high resolution complexes (<2.5 Å) of the antichlamydial antibodies S25-2, S73-2 and S25-39 with poly-Kdo (ketodeoxyoctulosonic acid) antigens were obtained from the Protein Data Bank (PDB) (Table 1). The test systems examined (anti-ganglioside antibodies and their ligands) are summarized in Table 2. Antibodies were numbered and CDRs defined following the IMGT unique numbering scheme [39].

### Molecular docking

Glide 5.6 [40], [41], GOLD 4.1.1 [42], AutoDock 4.2 [43] and DOCK 6.4 [44] were evaluated for their ability to reproduce the crystallographic binding mode in each of the validation systems. The settings used for these programs are detailed elsewhere [34], [45]. Briefly, the ligands were treated flexibly by each docking program, with the exception of the pyranose rings, which were kept in chair conformations. In the validation cases, all crystallographic waters and buffer molecules, as well as ions, were removed from the structures. The Protein Preparation Wizard in Maestro 9.2 [46] was used to add hydrogens and determine the most likely protonation states of titratable protein residues in all cases.

### Site mapping

The site mapping procedure was applied to the test systems as described previously [32]. Briefly, the interactions taking place in the top 100 ranked poses obtained from molecular docking are tallied according to the protein residue with which they occurred and the type of interaction taking place (i.e., hydrogen bond or van der Waals interaction). The tallies are normalized by dividing the number of interactions observed with a particular residue by the total number of interactions observed. Normalization is performed separately for hydrogen bonding and van der Waals interactions. The normalized tallies are sorted from greatest to least contribution, and the cumulative sum is calculated. All residues which occur above a given cumulative sum cutoff are deemed important for recognition.

The metrics of reproduction and correctness were used in the assessment of site map quality. Reproduction is calculated as the number of crystallographic interactions identified by the site map divided by the number of crystallographic interactions observed. Correctness is calculated as the number of crystallographic interactions identified by the site map divided by the total number of mapped interactions. Reproduction and correctness values close to one indicate that the generated site maps accurately identify the crystallographic interactions, without the inclusion of erroneous contacts. The product of reproduction and correctness was used to assess the quality of mapping; larger values indicate optimal reproduction and correctness. The cumulative sum cutoff was optimized for the validation systems by assessing the average product of reproduction and correctness at 10% cutoff intervals from 0–100%. The cutoff which provided the greatest average product for the series of validation cases (Table 1) was applied to investigate the recognition of gangliosides and ganglioside-mimetic peptides by the selected anti-ganglioside antibodies (Table 2).

### Dynamic site mapping

Three residues of the HCDR3 loop are missing from the structure of chP3 (PDB 3IU4) [30]. The missing portion was manually built, and low energy conformers of this portion, as well as the residue adjacent to each side of the missing portion (making a total of five residues), were generated using the loop refinement tool in Prime [47]. The site mapping procedure (see above) was performed on the ten lowest energy conformers. The most likely conformer from the set was selected on the basis of its similarity to the ensemble average of the hydrogen bonding and van der Waals site maps. The ensemble average maps were calculated by averaging the interaction contributions of each mapped residue across the set of ten conformers. The similarity of each conformer's site maps to the ensemble average was determined using the following expression:
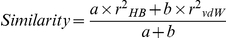
where *a* is the cutoff for selection of important hydrogen bonding contacts (as a fraction), *b* is the cutoff for selection of important van der Waals contacts (as a fraction), *r^2^_HB_* is the correlation coefficient calculated between a particular conformer and the ensemble average for hydrogen bonding contacts, and *r^2^_vdW_* is the correlation coefficient calculated between a particular conformer and the ensemble average for van der Waals contacts. The conformer exhibiting the highest similarity to the ensemble average maps was selected as the most likely conformer.

### Peptide mimicry of gangliosides

Ganglioside-mimetic peptides were separated into overlapping hexapeptide fragments and docked to the antibody targets using GOLD. The site mapping technique (see above) was applied to the resulting ensembles of poses. The interaction data for the set of hexapeptides was pooled to give one set of site maps for the complete peptides, as described earlier [36].

### Comparison of ganglioside and mimic recognition

To compare the recognition of gangliosides and their peptide-based mimics, scatter plots comparing the interaction contributions of antibody residues in ganglioside recognition and mimic recognition were generated. The distance between each point and the line representing equivalence of ganglioside and mimic recognition (*d*) was computed as described previously [36]. Positive *d* values indicate a greater number of interactions by that residue with the mimic, while negative *d* values indicate more interactions with the ganglioside. Residues with *d* greater than an absolute value of 3.00 were considered to vary significantly from the equivalence line.

## Results

### Molecular docking evaluation

**Figure 2:**
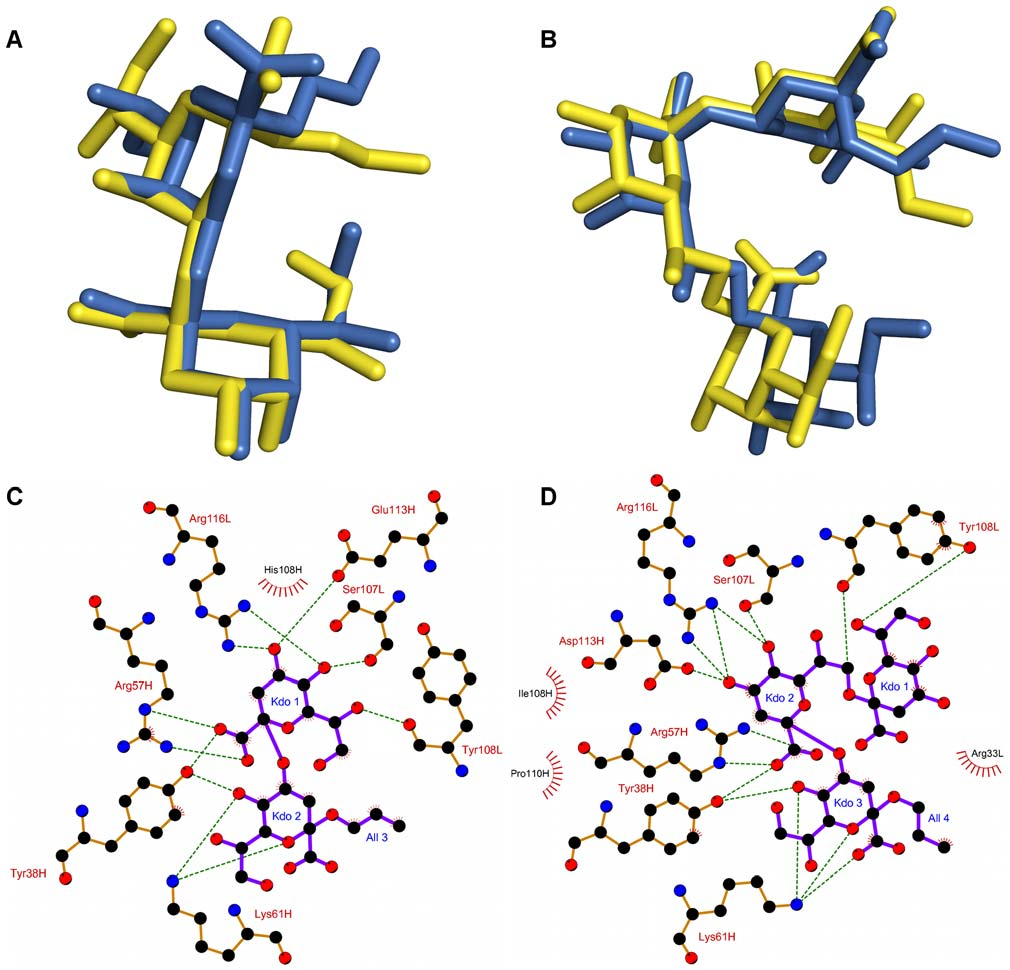
Evaluation of molecular docking using high resolution crystal structure complexes. A. Comparison of top ranked pose obtained from molecular docking (yellow) with the crystallographic binding mode (blue) of the Kdoα(2→4)Kdoα(2-OAll):S25-39 complex (PDB 3OKK). B. Comparison of top ranked pose (yellow) obtained from molecular docking with the crystallographic binding mode (blue) of the Kdoα(2→8)Kdoα(2→4)Kdoα(2-OAll):S73-2 complex (PDB 3HZV). C. Schematic representation of interactions in the Kdoα(2→4)Kdoα(2-OAll):S25-39 predicted by molecular docking. D. Schematic representation of interactions in the Kdoα(2→8)Kdoα(2→4)Kdoα(2-OAll):S73-2 complex. Molecular docking carried out using GOLD 4.1.1. Figures 2A and 2B prepared using PyMOL [64]. Figures 2C and 2D prepared using LIGPLOT [65]. Legend to Figures 2C and 2D: hydrogen bonds – green dashes; hydrophobic interactions – red arcs; carbon – black; oxygen – red; nitrogen – blue; ligand bonds – purple; protein bonds – orange.

Several molecular docking programs were evaluated for their ability to predict the crystallographic binding mode of a series of antichlamydial antibodies in complex with poly-Kdo antigens (Table 1). The results of molecular docking evaluation demonstrate that most programs are generally unsuccessful in accurately ranking the crystallographic binding mode (Table 3). However, all of the programs were able to identify the correct binding mode (i.e., less than 2.0 Å rmsd between pose and crystallographic binding mode) for at least one case, regardless of ranking. The exception to this is GOLD, which was able to both accurately identify and rank, as the top pose, the correct binding mode in four cases, these being all of the S73-2 complexes (PDB codes 3HZK, 3HZV and 3HZY), and the complex of S25-39 with Kdoα(2→4)Kdoα(2-OAll) (PDB 3OKK). Two of these successful cases are shown in Figure 2. In general, increasing the size and flexibility of the carbohydrate determinant being examined led to reduced quality predictions. Furthermore, the binding site topography may also impact on the quality of predictions, as observed previously [45], however, too few appropriate model complexes are available to confirm this.

**Table 3:** Molecular docking of validation systems.

	rmsd values (Å)							
	Glide		GOLD		Autodock		DOCK	
PDB code	top^a^	best^b^	top	best	top	best	top	best
1Q9Q	6.5	3.7 (15)	6.8	4.3 (2)	6.3	3.7 (27)	9.8	5.7 (7)
1Q9T	4.8	1.5 (7)	3.4	1.0 (5)	7.3	1.9 (3)	5.0	2.0 (4)
3HZK	4.9	1.3 (12)	1.6	1.5 (7)	3.5	3.4 (10)	4.6	1.3 (11)
3HZV	5.0	3.0 (4)	1.1	1.1 (1)	6.6	3.1 (81)	-c	-c
3HZY	8.4	1.2 (11)	1.5	1.2 (4)	8.6	3.2 (48)	8.1	6.3 (3)
3OKK	6.6	1.0 (11)	1.2	1.2 (1)	1.9	1.9 (1)	5.5	3.6 (11)
3OKL	5.8	3.3 (15)	6.6	0.8 (2)	3.6	1.5 (2)	5.5	4.6 (6)
3OKN	8.2	1.7 (11)	3.0	1.3 (8)	7.5	4.1 (30)	6.4	6.4 (1)
3OKO	7.1	2.1 (62)	6.2	3.5 (58)	4.5	3.8 (46)	-c	-c
Mean±S.D.	6.4±1.4	2.1±1.0	3.5±2.4	1.8±1.2	5.5±2.2	3.0±0.9	6.4±1.9	4.3±2.1

a Pose ranked first by molecular docking program.

b Pose which gave the best RMSD value compared to the crystallographic binding mode. The docking rank is shown in parentheses.

c No ligand poses were obtained following the docking procedure.

### Optimization of site mapping for antibody recognition of acidic sugars

Since GOLD produced the most accurate poses, irrespective of the ability to rank those poses, it was used to provide the pose ensemble input for site mapping. A cumulative sum cutoff of 80% for both hydrogen bonding and van der Waals interactions has been shown to be optimal when site mapping anti-carbohydrate antibodies, where shorter, less flexible and less functionally diverse carbohydrates were considered [32]. This cutoff has also been successfully applied to peptide-recognizing antibodies and carbohydrate-lectin interactions [34], [36]. In order to determine whether this cutoff was appropriate for studying antibody recognition of acidic sugars with highly flexible linkages (i.e., (1→6) or (2→8) linkages), a range of cutoff values was investigated (Table S1). The 90% cutoff was found to be most consistent, affording a lower standard deviation in the product data (i.e., reproduction×correctness) obtained for the set of cases (S.D. = 0.05). The 80% cutoff afforded an identical mean to the 90% cutoff across the set of studied cases, but was slightly less consistent than the 90% cutoff (S.D. = 0.08). However, the use of the 90% cutoff resulted in relatively poor map correctness (∼0.5–0.6) compared to previous cases [32], [34], [36]. This low level of map correctness can be attributed to the inclusion of many erroneous van der Waals contacts.

In order to optimize the selection of interacting residues, hydrogen bonds and van der Waals contacts were considered with separate cutoffs, instead of the same cutoff for each interaction type as previously used [32], [34], [36]. When hydrogen bonding was considered alone, the 90% cutoff was found to be optimal for the range of test systems (Table S2) and gave superior results to considering both hydrogen bonding and van der Waals with the same cutoff. Consideration of some van der Waals contacts is needed to identify interactions with non-polar sidechains. It was found that a 40% cutoff for van der Waals contacts, in combination with a 90% cutoff for hydrogen bonding interactions, provided the optimal prediction of crystallographic contacts (Table S3).

**Figure 3:**
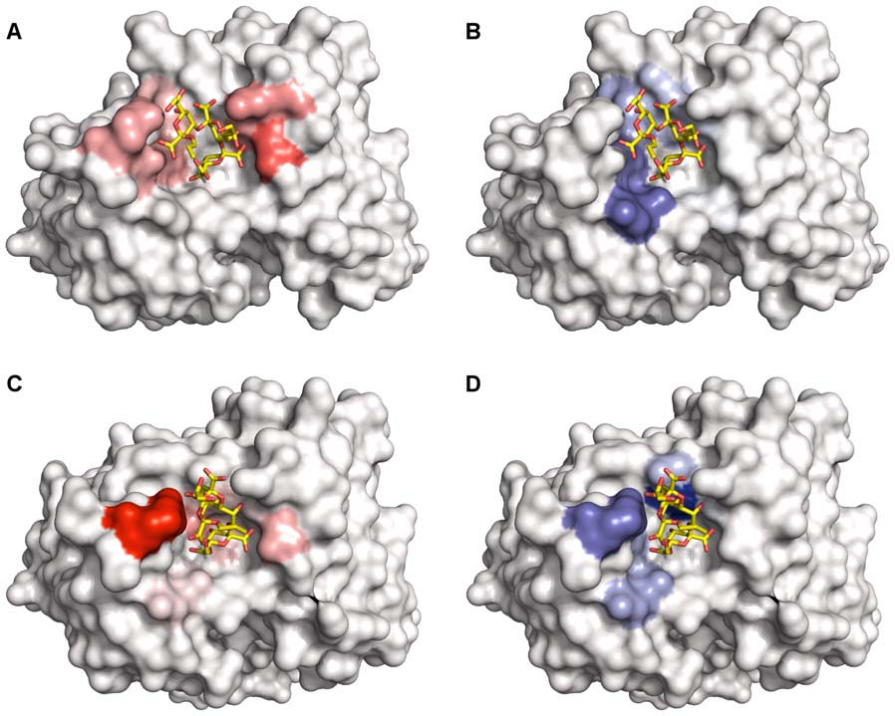
Evaluation of site mapping using high resolution crystal structure complexes. Kdoα(2→4)Kdoα(2→4)Kdoα(2-OAll) binding to S73-2 (PDB 3HZY) described by hydrogen bonding map (A) and van der Waals interaction map (B). Kdoα(2→8)Kdoα(2→4)Kdoα(2-OAll) binding to S25-39 (PDB 3OKO) described by hydrogen bonding map (C) and van der Waals interaction map (D). The color depth indicates the level of involvement of a particular residue in ligand recognition; more strongly illuminated residues are more involved in ligand recognition than weakly illuminated residues. The crystal structure binding mode is shown in each structure in sticks colored by atom type (C, yellow; O, red). Images rendered using PyMOL [64].

Using this optimized cutoff, it was demonstrated that site mapping and the top pose obtained from molecular docking performed comparably at the prediction of interacting residues (Figure 3, Table 4). Furthermore, this optimized cutoff affords site map quality comparable in reproduction and correctness to previously studied antibody- and lectin-carbohydrate systems [32], [34].

**Table 4:** Comparison of predictive ability of the optimized site mapping procedure and molecular docking.

	Reproduction^a^		Correctness^b^	
PDB code	Site map	Top pose	Site map	Top pose
1Q9Q	0.88	0.88	0.64	0.88
1Q9T	1.00	1.00	0.80	0.80
3HZK	1.00	1.00	0.67	0.73
3HZV	1.00	1.00	0.75	0.90
3HZY	0.80	0.50	0.73	0.71
3OKK	0.86	1.00	0.55	0.88
3OKL	0.78	0.78	0.78	0.78
3OKN	1.00	1.00	0.67	0.80
3OKO	1.00	0.89	0.75	0.88
Mean ± S.D.	0.92±0.09	0.89±0.17	0.70±0.08	0.82±0.07

a Computed as the number of interacting residues correctly identified by the technique divided by the total number of interacting residues in the crystallographic complex.

b Computed as the number of interacting residues correctly identified by the technique, divided by the total number of interacting residues identified by the technique.

### Dynamic site mapping of chP3

The structure of chP3 (PDB 31U4) is missing three residues from the HCDR3 loop [30]. It is known that residues from this loop are important for antigen recognition by chP3. In order to identify the most appropriate conformation of this loop, “dynamic” site mapping of chP3, whereby site mapping of multiple chP3 conformers – varying only in the conformation of the missing portion of the HCDR3 loop – was carried out. Since less than ten poses were obtained when docking Neu5Gc-GM3 to the fourth-lowest energy conformer of the chP3 HCDR3, site mapping could not be carried out on this structure. The eleventh-lowest energy conformer was used instead, to make a set of ten structures.

**Figure 4:**
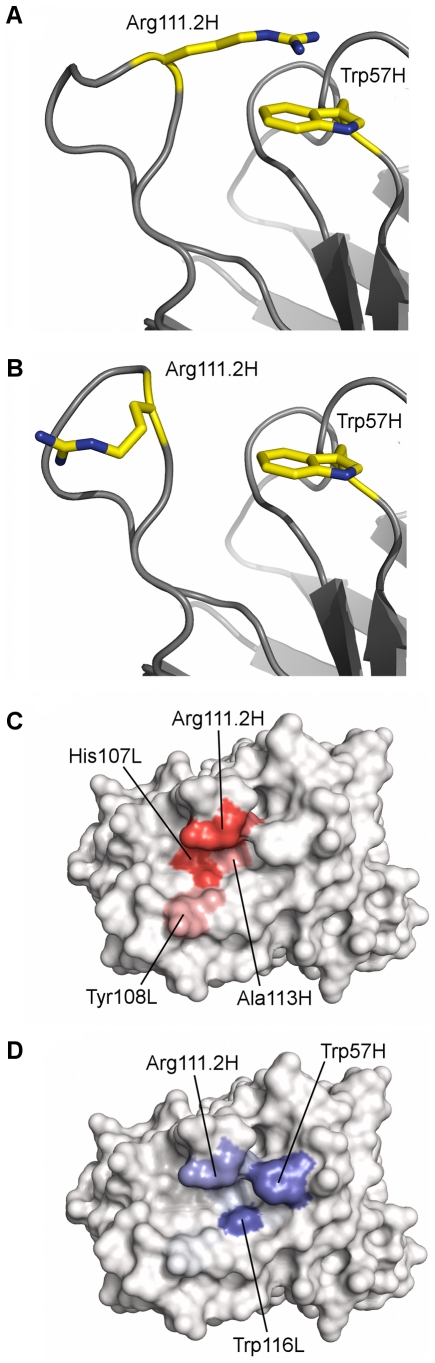
Dynamic mapping of chP3. A. The second-lowest energy conformer, highlighting the stacking between the side-chains of Arg111.2H and Trp57H. B. The tenth-lowest energy conformer, predicted to be most likely to be involved in ligand binding. C. Hydrogen bonding site map for tenth-lowest energy conformer. D. The van der Waals map for tenth-lowest energy conformer. Residues contributing to the proposed ganglioside-binding motif are highlighted on the hydrogen bonding site maps.

From site-directed mutagenesis studies, it is known that Arg111.2H (Arg100AH in Kabat numbering) is critical for ganglioside recognition [30]. It would therefore be expected that this residue is heavily involved in antigen interactions. When the site mapping procedure was carried out on the lowest energy chP3 HCDR3 conformer, Arg111.2H accounted for only 5.31% of all observed hydrogen bonds, while other residues accounted for a significantly greater number of hydrogen bonds (Table S4). Thus, the top scoring conformer is potentially not the most representative of the biologically relevant state. Similar site maps to this were observed for the fifth- and ninth-lowest energy chP3 structures. The second-lowest energy chP3 HCDR3 conformer featured almost no interactions with Arg111.2H (0.90% of hydrogen bonds and 1.34% of van der Waals interactions), suggesting that the loop is also likely to be in a biologically irrelevant conformation. Upon inspection of this structure, the side-chain of Arg111.2H stacks with the side-chain of Trp57H, and thus cannot easily interact with the ligand (Figure 4). In the third-lowest energy structure, the most hydrogen bonds were observed with Arg111.2H – just over 20% of all hydrogen bonds. Therefore, this structure is likely to be representative of the biologically relevant state. Other key contacts in this structure included Ala112.1H, His107L and Tyr108L. The sixth-lowest energy structure featured Arg111.2H and Ala112.1H as key contacts, but does not prominently feature the two residues from the light chain, identified as important for interactions in the third-lowest energy structure. Instead, Ser38H was identified as a key contact. The eighth-lowest energy structure was similar to this, with slightly greater emphasis on interactions with His107L and Tyr108L. In the seventh-lowest energy structure, Arg111.2H dominated the hydrogen bonding interactions, accounting for almost one third of all hydrogen bonds observed with that structure. However, Ala112.1H, Gln112H, His107L and Tyr108L each accounted for ∼10% of all hydrogen bonds. The tenth- and eleventh-lowest energy structures afforded similar site maps to one another, with hydrogen bonds fairly evenly distributed between Arg112.2H, Ala112.1H, Gln112H, Ala113H, His107L and Tyr108L.

In all cases, van der Waals contacts were dominated by Trp57H and Trp116L (Table S5). The HCDR3 residues – Arg111.2H, Ala112.1H and Gln112H – were also important for van der Waals contacts. Their importance is usually in line with their importance for hydrogen bonding (i.e., residues that are strongly important for hydrogen bonding are typically strongly important for van der Waals contacts).

In analyzing the generated site maps, it was determined that the chP3 conformer most representative of the average state was the tenth-ranked conformer (Figure 4, Table 5). This conformer features significant hydrogen bonding with Arg111.2H, known to be important for recognition from site-directed mutagenesis studies, but also suggests the importance of nearby HCDR3 residues (Ala112.1H, Gln112H, Ala113H) and the LCDR3 residues His107L and Tyr108L. The van der Waals interactions largely occur with tryptophan residues at positions 57H and 116L. This residue utilization is similar to other anti-carbohydrate antibodies [32].

**Table 5:** Conformer score for lowest energy conformers of chP3.

Conformer	*r^2^_HB_*	*r^2^_VdW_*	Similarity
1	0.69	0.70	0.69
2	0.50	0.59	0.53
3	0.89	0.57	0.79
5	0.66	0.55	0.62
6	0.53	0.40	0.49
7	0.70	0.55	0.65
8	0.72	0.58	0.68
9	0.75	0.79	0.76
10	0.95	0.63	0.85
11	0.87	0.69	0.82

### Determination of the likely ganglioside-recognizing motif

We had investigated ganglioside recognition by the four anti-ganglioside antibodies (Table 2) using identical hydrogen bonding and van der Waals cutoffs, as per our previous work [20], [32], [34], and this identified some of the residues likely to be involved in ganglioside recognition [48]. To confirm the results of this preliminary study, these cases have now been re-examined using the optimized cutoff values. The corresponding carbohydrate epitopes of the gangliosides were docked to R24, ME36.1 and 14F7, and the optimized cutoffs were applied to identify likely antibody residues involved in ganglioside recognition. The generated site maps, as well as that generated by the dynamic mapping procedure applied to chP3, were used to identify the presence of a potential ganglioside-binding motif in the anti-ganglioside antibodies. The key residues involved in ganglioside recognition are summarized in Table 6.

**Table 6:** Ganglioside recognition by test systems.^a^

		R24			ME36.1			14F7			chP3^b^		
Region	Position	Residue	%HB	%VdW	Residue	%HB	%VdW	Residue	%HB	%VdW	Residue	%HB	%VdW
HCDR1	35							Thr	5.42	1.31			
	36	Asn	2.60	2.87				Ser	14.19	16.34			
	37				Tyr	0.22	3.69	Tyr	4.01	14.71			
	38	Gly	7.99	3.98	Thr	13.90	7.78	Trp	10.46	10.78			
	40				His	4.71	3.96				His	3.69	3.42
HCDR2	55^c^	Tyr	10.39	3.98	Asp	4.60	5.67				Met	3.69	6.05
	57	Ser	1.60	5.10	Asn	1.01	2.64	Asp	15.50	8.66	Trp	0.78	14.87
	58	Ser	18.28	3.18									
	62							Thr	4.67	3.10			
	64	Ser	4.90	4.30									
	66^c^	Asn	2.80	0.48	Asn	4.48	1.72				Asp	2.46	1.05
HCDR3	107	Gly	0.10	11.15	Lys	2.47	6.46				Ser	2.80	0.66
	108	Gly	2.80	3.18	Ser	22.20	14.12						
	109	Thr	13.09	10.35									
	110	Gly	2.90	1.59				Arg	18.86	10.46			
	111	Thr	0.60	3.50									
	111.1	Arg	0.80	6.60									
	111.2							Arg	6.82	3.76	Arg	15.66	12.11
	112.1	Ser	8.79	10.51				Tyr	12.51	11.11	Ala	12.86	8.82
	112	Leu	5.49	3.50							Gln	16.11	10.26
	113	Tyr	13.59	16.24							Ala	10.07	7.11
	114	Tyr	0.30	3.18									
LCDR1	38				Asn	3.48	3.69						
	40^c^				His	2.69	1.85						
LCDR2	52				Leu	0.00	6.60						
	55^c^				Tyr	4.82	12.01						
	56^c^				Ser	7.06	2.11						
LCDR3	107				Arg	15.92	4.88				His	15.55	5.00
	108										Tyr	9.17	5.53
	114				Tyr	7.06	3.69						
	116										Trp	0.11	15.39

a Percentage contribution to interactions shown only for residues identified as important for recognition by site mapping.

b Results shown for HCDR3 conformer selected by dynamic site mapping.

c By definition, these positions occur in CDR-adjacent framework regions.

**Figure 5:**
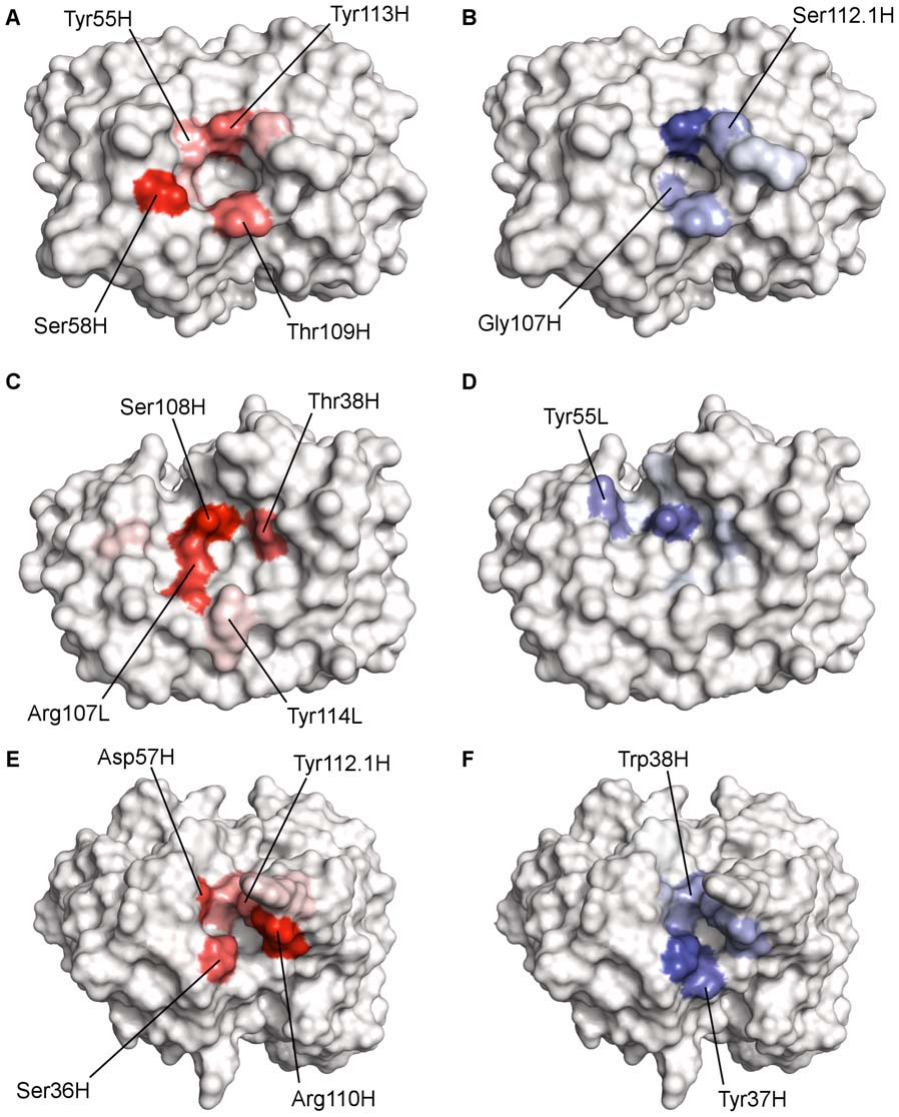
Site maps of anti-ganglioside antibodies. Hydrogen bonding and van der Waals site maps of R24 (A and B), ME36.1 (C and D) and 14F7 (E and F). Residues contributing to the likely ganglioside-binding motif are labeled on the hydrogen bonding site maps.

Ganglioside recognition by the antibodies was generally dominated by interactions with the heavy chain (Figures 4 and 5). In the cases of mAbs R24 and 14F7, recognition was entirely dependent on heavy chain residues, while for mAbs ME36.1 and chP3, approximately one third of all interactions occurred with light chain residues. These differences in CDR utilization can be explained in terms of the binding site topographies of each of the antibodies; the binding cavities of R24 and 14F7 are both comprised entirely of heavy chain residues, with access to the LCDRs blocked by HCDR2.

Despite the differences in binding site topographies, there are key similarities between the antibodies which become apparent upon structural examination of the site maps. Four residues, arranged in a relatively similar “spiral” around each antibody binding site, are largely responsible for hydrogen bonding interactions with the gangliosides. Proceeding clockwise, the likely ganglioside-binding motif of the antiganglioside antibodies comprises two polar residues (typically Ser, followed by Tyr, Thr or Asp), an aromatic residue (typically Tyr) and a basic residue (Arg). Not all of the antibodies strictly conform to this motif; for example, R24 features a threonine residue where an arginine would be expected, and chP3 features an alanine residue where a serine would be expected.

### Peptide mimicry of gangliosides

**Figure 6:**
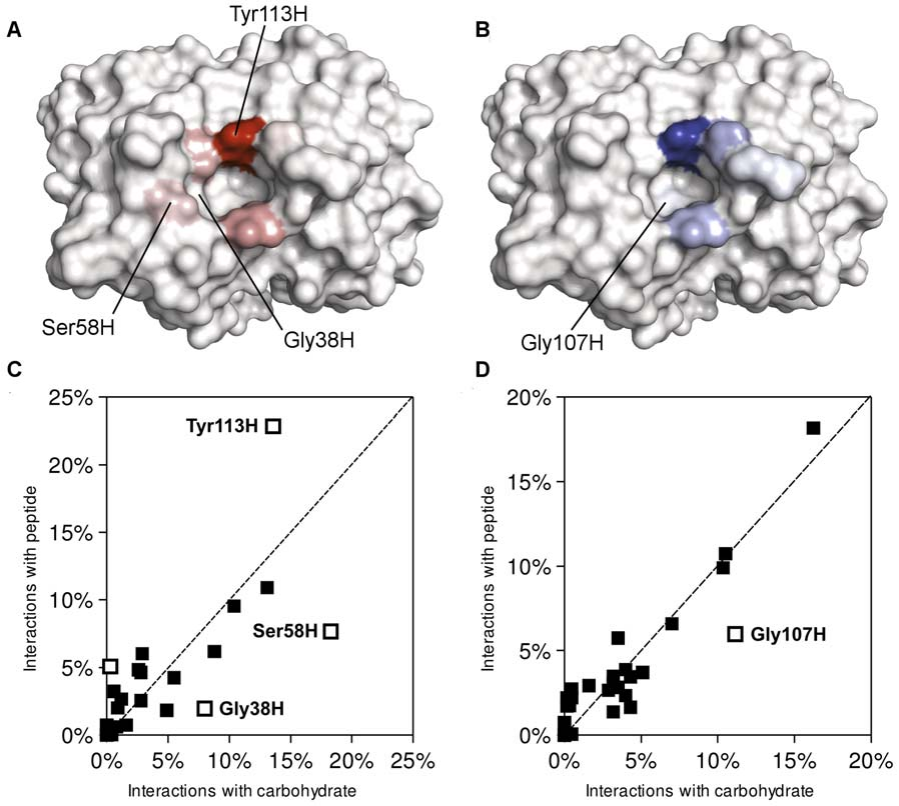
Peptide mimicry of GD3 binding to R24. A. Hydrogen bonding site map describing peptide (RHAYRSMAEWGFLYS) recognition by R24. B. van der Waals interaction site map describing peptide recognition by R24. Site maps generated and rendered using PyMOL [64]. C. Comparison of hydrogen bonding site maps describing GD3 and peptide recognition. D. Comparison of van der Waals site maps describing GD3 and peptide recognition. In Figures 6C and 6D, open points indicate residues which deviate significantly from the line representing equivalence of carbohydrate and peptide interactions (i.e., |*d*|>3.00). The open point not labeled on Figure 6C corresponds with Tyr114H.

The peptide mimic of GD3 – RHAYRSMAEWGFLYS – could replicate almost all of the van der Waals interactions made by the ganglioside with R24, but some marked differences in the hydrogen bonding profile occurred (Table S6). The maps revealed that the peptide failed to replicate the number of hydrogen bonds made between the GD3 trisaccharide and the antibody residues Gly38H and Ser58H. However, a significant increase in hydrogen bonds with the HCDR3 residues, Tyr113H and Tyr114H, was observed. The reduced potential of the peptide to deeply and consistently penetrate the binding site across the pose ensemble may explain why fewer interactions were observed with Gly38H and Ser58H. Tyr113H and Tyr114H may afford more interactions with the peptide, since they are more easily accessible. From comparison of the ganglioside- and peptide-derived site maps (Figure 6), the peptide appears to be a partial structural mimic of GD3 [20]. This partial structural mimicry could account for the observed immunological mimicry of GD3 by this peptide [25].

**Figure 7:**
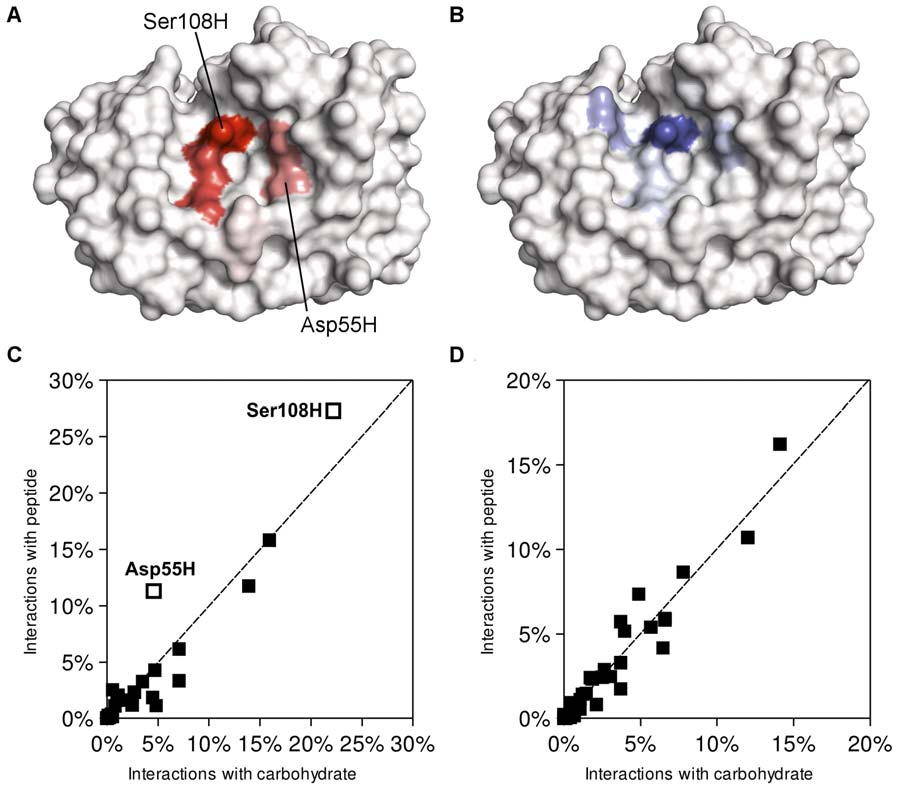
Peptide mimicry of GD2 binding to ME36.1. A. Hydrogen bonding site map describing peptide (LDVVLAWRDGLSGAS) recognition by ME36.1. B. van der Waals interaction site map describing peptide recognition by ME36.1. C. Comparison of hydrogen bonding site maps describing GD2 and peptide recognition. D. Comparison of van der Waals site maps describing GD2 and peptide recognition.

The peptide mimic of GD2 could more closely mimic carbohydrate binding to ME36.1 compared to the peptide mimic of GD3 binding to R24 (Table S7). Similar to carbohydrate and peptide binding to R24, the van der Waals interactions of the carbohydrate were very well replicated by the peptide, while some minor differences in the hydrogen bonding profile were observed. A significant increase in hydrogen bonds with Asp55H and Ser108H was observed, accompanied by a slight drop in hydrogen bonds with Tyr55L and Ser56L. Despite these differences, the ganglioside- and peptide-derived site maps for ME36.1 are overall very similar to one another. Since the peptide is known to be an immunogenic mimic of GD2 [23], this represents a case of structural mimicry translating into immunological mimicry (Figure 7) [20].

## Discussion

Carbohydrate-protein recognition is particularly challenging for molecular docking to predict accurately, due to the multitude of chemically equivalent hydroxyl groups in carbohydrates and their potential to form many specific interactions, including CH-π interactions [45], [49]. Evaluation of a variety of molecular docking approaches against suitable test cases is desirable to ensure the development of an optimal modeling protocol. We have previously demonstrated that molecular docking can be an effective tool for studying antibody recognition of structurally simpler carbohydrates [45]. However, gangliosides feature diverse chemical functionality, including carboxylate groups and flexible hydroxylated chains. Furthermore, GD2 and GD3 contain α(2→8) linkages, which feature an additional degree of conformational freedom compared to, for example, (1→3) and (1→4) linkages, which we have previously investigated [45]. To our knowledge, such systems have not been appropriately evaluated with molecular docking. While high resolution ganglioside-antibody complexes are not available for use in method evaluation, a series of high resolution structures of antichlamydial antibodies in complex with Kdo-containing carbohydrates are available [50], [51], [52] (Table 1); these represent the most suitable model systems for evaluating the likelihood of success in predicting ganglioside-antibody recognition. Another issue with molecular docking is the need to consider the potential of multiple ligand binding modes. Their effect on recognition by proteins may be important for highly flexible ligands, such as carbohydrates and peptides [49]. In order to consider the effect of multiple ligand binding modes on recognition by proteins [53], we developed the site mapping technique, which we have demonstrated to be effective for studying carbohydrate-antibody [32] and carbohydrate-lectin recognition [34], as well as carbohydrate-peptide mimicry [36].

In this study, the site mapping methodology is extended to study the recognition of acidic sugars, particularly gangliosides, by antibodies. Furthermore, to study ganglioside recognition by chP3, a “dynamic” version of the site mapping technique was developed, whereby multiple antibody conformers are considered to select one with the most likely ligand-binding conformation. Ganglioside mimicry is investigated by comparing the site maps describing the recognition of gangliosides and their peptide-based mimics.

All of the antibodies examined in the validation set (Table 1) prominently feature arginine residues in their binding sites, which pair with the carboxylate groups of the complexed carbohydrate. Since these charge-assisted hydrogen bonds are likely to be highly energetically favorable, it was anticipated that the pairing effect would lead to good quality predictions of antibody recognition of acidic sugars. The pairing effect gives rise to good quality predictions in GOLD for four of the validation cases. However, increasing both the number of residues as well as the flexibility of the carbohydrate (i.e., including one or more α(2→8) linkages) generally results in reduced accuracy. This result is not surprising, as these factors are known to affect the accuracy of molecular docking [49]. Furthermore, the carbohydrates in the validation cases contain multiple carboxylate groups, therefore, it is possible that the incorrect carboxylate group could be paired against a specific arginine residue, particularly if the rest of molecule can be accommodated in the binding site and favorably scored. This is not an issue for the majority of the test cases (Table 2), which feature only one carboxylate group. Since only a limited number of antibodies are available for use as validation cases, the effect of binding site topography on docking accuracy could not be effectively studied, as it had been previously [34], [45]. However, the case of 3HZV, which contains a large, highly flexible carbohydrate, suggests that subtle changes in binding site topography have a dramatic impact on docking accuracy.

In our previous site mapping studies, identical cutoffs of hydrogen bonding and van der Waals interactions were used to select residues involved in ligand recognition [32], [34], [36]. However, the use of identical cutoffs for both of these interaction types does not result in an optimal model for gangliosides. A significant bias towards hydrogen bonding interactions is needed to accurately represent antibody recognition of acidic sugars. Furthermore, consideration of van der Waals interactions resulted in only a slight improvement in the overall accuracy of the site mapping procedure. This suggests that hydrogen bonding interactions are significantly more important for antibody recognition of acidic sugars than van der Waals interactions. Systems previously studied *via* site mapping [32], [34], [36] may benefit from this “mixed” treatment of hydrogen bonding and van der Waals interactions.

The dynamic mapping study of chP3 demonstrates that the site mapping technique has applications not just in identifying antibody residues important for ligand interactions, but also in identifying the likely bioactive conformation of CDR loops, particularly HCDR3, which is often crucial for antigen recognition and for which no canonical structures exist [54], [55]. Importantly, the selected protein conformer did not correspond with the lowest energy state identified, suggesting that, at least in the case of chP3, conformational change upon antigen binding may be important in recognition. The role of conformational change and induced-fit mechanisms in antigen binding has been explored earlier by a number of groups [56], [57], [58], [59], [60]. The dynamic mapping study highlights the potential usefulness of the site mapping technique in the homology modeling of antibodies and in the structural elucidation of conformational change upon antigen binding.

Although the four anti-ganglioside antibodies studied have quite distinct binding site topographies and varying specificities (Table 2), enough similarities in residue utilization across the set could be identified to propose a potential ganglioside-binding motif. This suggests the possibility of structural convergence in the immune response towards a given antigen class. Structural convergence has been reported earlier for anti-Lewis Y antibodies [61]. It is likely that anti-ganglioside antibodies specifically recognize terminal sialic acid residues, since these are the most accessible, and thus, antibodies may consistently contain a particular sialic acid-binding motif. Structural analysis of additional anti-ganglioside antibodies is required in order to evaluate this hypothesis. The currently identified motif provides a potential framework for optimizing existing anti-ganglioside antibodies.

In comparing ganglioside and peptide recognition, we were able to determine that peptides known to act as immunological mimics of gangliosides could also act at least as partial structural mimics of the gangliosides. Therefore, it may be possible to design peptides which are capable of inducing an anti-ganglioside immune response using the antibody site maps generated in this study. Such a “design by mapping” procedure was proposed in our earlier work in investigating peptide mimicry of αGal-terminating carbohydrates [36]. Our results for docking anti-idiotypic antibodies to anti-Neu5Gc-GM3 antibodies indicate that anti-idiotypic antibodies do not act as structural mimics of gangliosides. This is most likely due to protein-protein recognition involving a more extensive interaction network compared with protein-carbohydrate association.

While the site mapping technique solely evaluates ligand recognition from the protein's “point-of-view”, it can be combined with ligand-based mapping techniques to provide the complete picture of ligand-protein recognition [62]. Thus, this study not only suggested how gangliosides and their mimics are recognized by antibodies, but also raised several important new lines of inquiry relevant to future development of our mapping techniques. Studies are currently underway to answer these new questions, which will contribute to the robustness of the mapping techniques in probing carbohydrate-antibody recognition and to better understanding such recognition.

To summarize our findings, the anti-ganglioside antibodies studied here largely utilize a motif of four residues to recognize gangliosides (Ser, polar, aromatic, Arg). These residues are arranged within the binding site of each antibody studied here in a relatively similar fashion. Peptides which bind to anti-ganglioside antibodies and elicit anti-ganglioside immune responses were found to act as structural mimics of the gangliosides, providing a case where structural mimicry translates into immunological mimicry. Our findings provide structural details invaluable for the future development of ganglioside-targeting cancer vaccines or optimizing therapeutic antibodies, as well as demonstrating the potential role of the site mapping technique in structure-based vaccine design.

## Supporting Information

Table S1 **Initial optimization of site mapping cutoff using validation systems.**
(DOC)

Table S2 **Optimization of site mapping cutoff for hydrogen bonding using validation systems.**
(DOC)

Table S3 **Optimization of site mapping cutoff for van der Waals interactions using validation systems.**
(DOC)

Table S4 **Hydrogen bonding interactions in top ranked HCDR3 conformers of chP3.**
(DOC)

Table S5 **van der Waals interactions in top ranked HCDR3 conformers of chP3.**
(DOC)

Table S6 **Comparison of carbohydrate and peptide recognition by R24.**
(DOC)

Table S7 **Comparison of carbohydrate and peptide recognition by ME36.1.**
(DOC)
