# Supplementary material for: Antibody Recognition of Cancer-Related Gangliosides and Their Mimics Investigated Using in silico Site Mapping
Source: PLoS One. 2012 Apr 20;7(4):e35457. doi: 10.1371/journal.pone.0035457 (PMC3334985; doi:10.1371/journal.pone.0035457)
Supplement: Table S1 — Initial optimization of site mapping cutoff using validation systems. (DOC) [file pone.0035457.s001.doc]

Table S1. Initial optimization of site mapping cutoff using validation systems.

|  | **Reproduction × Correctness at cutoff** | | | | | | | | | |
| --- | --- | --- | --- | --- | --- | --- | --- | --- | --- | --- |
| **PDB code** | 10% | 20% | 30% | 40% | 50% | 60% | 70% | 80% | **90%** | 100% |
| 1Q9Q | 0.25 | 0.28 | 0.33 | 0.45 | 0.45 | 0.39 | 0.45 | 0.51 | **0.53** | 0.38 |
| 1Q9T | 0.25 | 0.28 | 0.40 | 0.52 | 0.64 | 0.56 | 0.56 | 0.56 | **0.62** | 0.44 |
| 3HZK | 0.06 | 0.28 | 0.28 | 0.52 | 0.52 | 0.68 | 0.56 | 0.51 | **0.50** | 0.44 |
| 3HZV | 0.22 | 0.44 | 0.44 | 0.36 | 0.35 | 0.49 | 0.45 | 0.55 | **0.60** | 0.39 |
| 3HZY | 0.20 | 0.23 | 0.27 | 0.36 | 0.54 | 0.49 | 0.45 | 0.53 | **0.51** | 0.42 |
| 3OKK | 0.29 | 0.32 | 0.46 | 0.51 | 0.45 | 0.40 | 0.47 | 0.58 | **0.50** | 0.35 |
| 3OKL | 0.22 | 0.33 | 0.25 | 0.46 | 0.46 | 0.50 | 0.40 | 0.49 | **0.55** | 0.53 |
| 3OKN | 0.06 | 0.17 | 0.40 | 0.33 | 0.45 | 0.50 | 0.45 | 0.47 | **0.50** | 0.35 |
| 3OKO | 0.22 | 0.22 | 0.33 | 0.36 | 0.40 | 0.50 | 0.65 | 0.75 | **0.60** | 0.43 |
| Mean | 0.20 | 0.28 | 0.35 | 0.43 | 0.47 | 0.50 | 0.49 | 0.55 | **0.55** | 0.41 |
| S.D. | 0.08 | 0.08 | 0.08 | 0.08 | 0.08 | 0.09 | 0.08 | 0.08 | **0.05** | 0.06 |
