# Supplementary material for: Antibody Recognition of Cancer-Related Gangliosides and Their Mimics Investigated Using in silico Site Mapping
Source: PLoS One. 2012 Apr 20;7(4):e35457. doi: 10.1371/journal.pone.0035457 (PMC3334985; doi:10.1371/journal.pone.0035457)
Supplement: Table S3 — Optimization of site mapping cutoff for van der Waals interactions using validation systems. (DOC) [file pone.0035457.s003.doc]

Table S3. Optimization of site mapping cutoff for van der Waals interactions using validation systems.^a^

|  | **Reproduction × Correctness at cutoff** | | | | | | | | | |
| --- | --- | --- | --- | --- | --- | --- | --- | --- | --- | --- |
| **PDB code** | 10% | 20% | 30% | **40%** | 50% | 60% | 70% | 80% | 90% | 100% |
| 1Q9Q | 0.56 | 0.56 | 0.56 | **0.56** | 0.56 | 0.51 | 0.47 | 0.57 | 0.53 | 0.38 |
| 1Q9T | 0.80 | 0.80 | 0.80 | **0.80** | 0.73 | 0.62 | 0.62 | 0.62 | 0.62 | 0.44 |
| 3HZK | 0.56 | 0.56 | 0.56 | **0.67** | 0.67 | 0.62 | 0.62 | 0.57 | 0.56 | 0.44 |
| 3HZV | 0.65 | 0.75 | 0.75 | **0.75** | 0.69 | 0.69 | 0.64 | 0.64 | 0.60 | 0.39 |
| 3HZY | 0.49 | 0.49 | 0.49 | **0.58** | 0.68 | 0.68 | 0.62 | 0.62 | 0.51 | 0.42 |
| 3OKK | 0.47 | 0.47 | 0.47 | **0.47** | 0.47 | 0.47 | 0.43 | 0.54 | 0.50 | 0.35 |
| 3OKL | 0.60 | 0.60 | 0.60 | **0.60** | 0.60 | 0.54 | 0.45 | 0.45 | 0.55 | 0.53 |
| 3OKN | 0.73 | 0.73 | 0.73 | **0.73** | 0.73 | 0.73 | 0.67 | 0.57 | 0.50 | 0.35 |
| 3OKO | 0.75 | 0.75 | 0.75 | **0.75** | 0.75 | 0.75 | 0.75 | 0.75 | 0.60 | 0.45 |
| Mean | 0.62 | 0.63 | 0.64 | **0.66** | 0.65 | 0.62 | 0.59 | 0.59 | 0.55 | 0.42 |
| S.D. | 0.12 | 0.12 | 0.12 | **0.11** | 0.09 | 0.10 | 0.11 | 0.08 | 0.05 | 0.06 |

^a^The hydrogen bonding site maps at the 90% cutoff were used as the starting point.
