# Supplementary material for: Antibody Recognition of Cancer-Related Gangliosides and Their Mimics Investigated Using in silico Site Mapping
Source: PLoS One. 2012 Apr 20;7(4):e35457. doi: 10.1371/journal.pone.0035457 (PMC3334985; doi:10.1371/journal.pone.0035457)
Supplement: Table S4 — Hydrogen bonding interactions in top ranked HCDR3 conformers of chP3. (DOC) [file pone.0035457.s004.doc]

Table S4. Hydrogen bonding interactions in top ranked HCDR3 conformers of chP3.a

|  | **Conformer rank^b^** | | | | | | | | | |  |
| --- | --- | --- | --- | --- | --- | --- | --- | --- | --- | --- | --- |
| **Residue** | **1** | **2** | **3** | **5** | **6** | **7** | **8** | **9** | **10** | **11** | **Avg** |
| Arg36H | 0.00 | 0.00 | 1.01 | 0.00 | 3.74 | 0.32 | 2.33 | 1.19 | 0.00 | 0.94 | 0.95 |
| Ser38H | 0.36 | 1.58 | 5.38 | 2.24 | 11.32 | 2.96 | 9.02 | 6.18 | 1.90 | 5.16 | 4.61 |
| His40H | 2.17 | 3.15 | 2.94 | 3.39 | 3.84 | 2.22 | 3.16 | 3.68 | 3.69 | 3.56 | 3.18 |
| Met55H | 3.38 | 4.05 | 2.13 | 4.17 | 4.32 | 2.65 | 3.07 | 5.20 | 3.69 | 3.41 | 3.61 |
| Asp66H | 4.10 | 4.73 | 1.62 | 2.71 | 2.98 | 2.75 | 2.14 | 4.01 | 2.46 | 0.94 | 2.84 |
| Ser107H | 0.72 | 2.25 | 2.84 | 2.62 | 5.57 | 2.75 | 4.65 | 5.09 | 2.80 | 4.14 | 3.34 |
| Gly108H | 0.00 | 0.11 | 1.12 | 0.10 | 1.92 | 0.95 | 2.23 | 2.28 | 0.11 | 2.40 | 1.12 |
| Arg110H | 1.69 | 0.79 | 0.00 | 3.68 | 0.00 | 0.00 | 0.00 | 0.11 | 0.22 | 0.73 | 0.72 |
| Glu111H | 3.38 | 0.00 | 0.94 | 1.36 | 0.00 | 0.00 | 0.00 | 0.00 | 0.00 | 1.45 | 0.71 |
| Gly111.1H | 0.36 | 9.01 | 0.00 | 2.03 | 2.30 | 0.11 | 2.14 | 0.43 | 0.00 | 0.00 | 1.64 |
| Arg111.2H | 5.31 | 0.90 | 22.62 | 5.04 | 24.66 | 32.91 | 24.74 | 5.31 | 15.66 | 16.41 | 15.36 |
| Ala112.1H | 16.04 | 15.77 | 13.59 | 15.70 | 17.27 | 9.63 | 15.26 | 16.03 | 12.86 | 8.06 | 14.02 |
| Gln112H | 17.85 | 18.02 | 9.03 | 19.67 | 5.47 | 8.25 | 7.53 | 15.28 | 16.11 | 10.53 | 12.77 |
| Ala113H | 8.93 | 8.33 | 5.68 | 7.85 | 5.28 | 5.40 | 5.49 | 10.73 | 10.07 | 8.64 | 7.64 |
| Trp114H | 1.21 | 0.34 | 0.00 | 0.48 | 0.00 | 0.21 | 0.19 | 0.22 | 0.45 | 1.23 | 0.43 |
| Thr37L | 0.97 | 0.90 | 0.41 | 1.26 | 0.00 | 0.32 | 0.00 | 0.43 | 0.89 | 1.23 | 0.64 |
| Ser56L | 3.98 | 2.93 | 1.83 | 5.52 | 0.10 | 1.16 | 0.09 | 1.19 | 2.01 | 2.47 | 2.13 |
| His107L | 17.49 | 15.65 | 14.00 | 13.66 | 2.11 | 13.33 | 6.70 | 12.68 | 15.55 | 15.11 | 12.63 |
| Tyr108L | 10.13 | 9.01 | 11.56 | 6.20 | 3.74 | 9.63 | 5.49 | 7.15 | 9.17 | 8.06 | 8.01 |
| *r^2^_HB_* | 0.69 | 0.50 | 0.89 | 0.66 | 0.53 | 0.70 | 0.72 | 0.73 | 0.95 | 0.87 | 1.00 |

^a^All values are percentages of the total number of hydrogen bonds observed. ^b^Less than ten ganglioside poses were generated for the fourth-lowest energy HCDR3 conformer of chP3. In order to consider a set of ten structures, the eleventh-lowest energy HCDR3 conformer was analyzed.
