# Supplementary material for: Antibody Recognition of Cancer-Related Gangliosides and Their Mimics Investigated Using in silico Site Mapping
Source: PLoS One. 2012 Apr 20;7(4):e35457. doi: 10.1371/journal.pone.0035457 (PMC3334985; doi:10.1371/journal.pone.0035457)
Supplement: Table S5 — van der Waals interactions in top ranked HCDR3 conformers of chP3. (DOC) [file pone.0035457.s005.doc]

Table S5. van der Waals interactions in top ranked HCDR3 conformers of chP3.a

|  | **Conformer rank** | | | | | | | | | |  |
| --- | --- | --- | --- | --- | --- | --- | --- | --- | --- | --- | --- |
| **Residue** | **1** | **2** | **3** | **5** | **6** | **7** | **8** | **9** | **10** | **11** | **Avg** |
| Trp57H | 15.34 | 15.59 | 16.39 | 14.62 | 20.71 | 16.15 | 18.40 | 17.59 | 14.87 | 12.43 | 16.21 |
| Arg111.2H | 2.76 | 1.34 | 13.80 | 1.78 | 14.42 | 13.93 | 13.25 | 4.75 | 12.11 | 7.05 | 8.52 |
| Ala112.1H | 9.20 | 9.81 | 8.61 | 11.20 | 13.84 | 6.38 | 11.61 | 12.58 | 8.82 | 7.98 | 10.00 |
| Gln112H | 8.90 | 9.95 | 4.48 | 9.43 | 4.00 | 4.82 | 4.05 | 8.60 | 10.26 | 9.55 | 7.40 |
| Trp116L | 16.26 | 16.40 | 13.21 | 14.48 | 9.61 | 14.97 | 11.61 | 15.02 | 15.39 | 12.62 | 13.96 |
| *r^2^_VdW_* | 0.70 | 0.59 | 0.57 | 0.55 | 0.40 | 0.55 | 0.58 | 0.79 | 0.63 | 0.69 | 1.00 |

^a^All values are percentages of the total number of van der Waals interactions observed.
