# Supplementary material for: Antibody Recognition of Cancer-Related Gangliosides and Their Mimics Investigated Using in silico Site Mapping
Source: PLoS One. 2012 Apr 20;7(4):e35457. doi: 10.1371/journal.pone.0035457 (PMC3334985; doi:10.1371/journal.pone.0035457)
Supplement: Table S6 — Comparison of carbohydrate and peptide recognition by R24. (DOC) [file pone.0035457.s006.doc]

Table S6. Comparison of carbohydrate and peptide recognition by R24.

|  | **Hydrogen bonding** | | | **van der Waals interactions** | | |
| --- | --- | --- | --- | --- | --- | --- |
| **Residue** | Carbohydrate^a^ | Peptide^a^ | *d* | Carbohydrate^b^ | Peptide^b^ | *d* |
| Asn36H | 2.60 | 4.83 | 1.58 | 2.87 | 2.65 | -0.15 |
| Gly38H | 7.99 | 1.96 | **-4.27** | 3.98 | 2.34 | -1.16 |
| Tyr55H | 10.39 | 9.54 | -0.60 | 3.98 | 3.88 | -0.07 |
| Ser57H | 1.60 | 0.73 | -0.62 | 5.10 | 3.70 | -0.99 |
| Ser58H | 18.28 | 7.67 | **-7.51** | 3.18 | 1.37 | -1.28 |
| Ser64H | 4.90 | 1.83 | -2.17 | 4.30 | 3.45 | -0.60 |
| Asn66H | 2.80 | 2.54 | -0.18 | 0.48 | 2.21 | 1.23 |
| Gly107H | 0.10 | 0.38 | 0.20 | 11.15 | 5.98 | **-3.65** |
| Gly108H | 2.80 | 4.63 | 1.30 | 3.18 | 3.07 | -0.08 |
| Thr109H | 13.09 | 10.92 | -1.54 | 10.35 | 9.90 | -0.32 |
| Gly110H | 2.90 | 6.02 | 2.21 | 1.59 | 2.93 | 0.94 |
| Thr111H | 0.60 | 3.24 | 1.86 | 3.50 | 2.82 | -0.48 |
| Arg111.1H | 0.80 | 0.60 | -0.14 | 6.60 | 7.01 | -0.29 |
| Ser112.1H | 8.79 | 6.19 | -1.84 | 10.51 | 10.74 | 0.16 |
| Leu112H | 5.49 | 4.25 | -0.88 | 3.50 | 5.75 | 1.59 |
| Tyr113H | 13.59 | 22.85 | **6.55** | 16.24 | 18.18 | 1.37 |
| Tyr114H | 0.30 | 5.08 | **3.38** | 3.18 | 3.48 | 0.21 |

^a^All values are percentages of the total number of hydrogen bonds made by that type of ligand. ^b^All values are percentages of the total number of van der Waals interactions made by that type of ligand.
