# Supplementary material for: Antibody Recognition of Cancer-Related Gangliosides and Their Mimics Investigated Using in silico Site Mapping
Source: PLoS One. 2012 Apr 20;7(4):e35457. doi: 10.1371/journal.pone.0035457 (PMC3334985; doi:10.1371/journal.pone.0035457)
Supplement: Table S7 — Comparison of carbohydrate and peptide recognition by ME36.1. (DOC) [file pone.0035457.s007.doc]

Table S7. Comparison of carbohydrate and peptide recognition by ME36.1.

|  | **Hydrogen bonding** | | | **van der Waals interactions** | | |
| --- | --- | --- | --- | --- | --- | --- |
| **Residue** | Carbohydrate^a^ | Peptide^a^ | *d* | Carbohydrate^b^ | Peptide^b^ | *d* |
| Tyr37H | 0.22 | 0.16 | -0.05 | 3.69 | 5.72 | 1.44 |
| Thr38H | 13.90 | 11.75 | -1.52 | 7.78 | 8.66 | 0.62 |
| His40H | 4.71 | 4.29 | -0.30 | 3.96 | 5.16 | 0.85 |
| Asp55H | 4.60 | 11.30 | **4.74** | 5.67 | 5.40 | -0.19 |
| Asn57H | 1.01 | 1.75 | 0.52 | 2.64 | 2.89 | 0.18 |
| Asn66H | 4.48 | 1.86 | -1.85 | 1.72 | 2.41 | 0.49 |
| Lys107H | 2.47 | 1.19 | -0.90 | 6.46 | 4.17 | -1.62 |
| Ser108H | 22.20 | 27.30 | **3.61** | 14.12 | 16.24 | 1.50 |
| Asn38L | 3.48 | 3.25 | -0.16 | 3.69 | 1.75 | -1.37 |
| His40L | 2.69 | 2.32 | -0.26 | 1.85 | 2.32 | 0.34 |
| Leu52L | 0.00 | 0.00 | 0.00 | 6.60 | 5.92 | -0.48 |
| Tyr55L | 12.01 | 10.71 | -2.61 | 4.82 | 1.12 | -0.92 |
| Ser56L | 7.06 | 3.34 | -2.63 | 2.11 | 0.81 | -0.92 |
| Arg107L | 15.92 | 15.82 | -0.07 | 4.88 | 7.35 | 1.75 |
| Tyr114L | 7.06 | 6.16 | -0.64 | 3.69 | 3.29 | -0.29 |
| Phe116L | 0.00 | 0.00 | 0.00 | 6.60 | 5.82 | -0.55 |

^a^All values are percentages of the total number of hydrogen bonds made by that type of ligand. ^b^All values are percentages of the total number of van der Waals interactions made by that type of ligand.
